# Numerical Investigation of the Performance of Segmental CFST Piers with External Energy Dissipators under Lateral Cyclic Loadings

**DOI:** 10.3390/ma15196993

**Published:** 2022-10-09

**Authors:** Chengquan Wang, Zheng Qu, Yonggang Shen, Jiqing Jiang, Chongli Yin, Yanwei Zong

**Affiliations:** 1 Department of Civil Engineering, Zhejiang University City College, Hangzhou 310015, China; 2 Zhejiang Engineering Research Center of Intelligent Urban Infrastructure, Hangzhou 310015, China; 3 Key Laboratory of Safe Construction and Intelligent Maintenance for Urban Shield Tunnels of Zhejiang Province, Hangzhou 310015, China; 4 School of Environment and Civil Engineering, Jiangnan University, Wuxi 214122, China; 5 Department of Civil Engineering, Zhejiang University, Hangzhou 310058, China

**Keywords:** segmental assembly of CFST piers, external energy dissipator, quasistatic analysis, seismic performance, bearing capacity formula

## Abstract

In order to improve the construction efficiency of piers and reduce the local damage of piers, concrete-filled steel tubes (CFST) are used to precast pier segments. Aiming at the problems of the poor integrity and insufficient energy dissipation capacity of dry joint segmental assembled piers, segmental assembled concrete-filled steel tubular piers with external replaceable energy dissipators are being developed. Based on the low cyclic test of a segmental assembled CFST pier, the finite element numerical simulation model of a CFST pier is established based on ABAQUS software, and the validity of the numerical model is verified by the experimental results. The effects of the section ratio, axial compression ratio, and initial prestress on the seismic performance of piers are studied through a pseudostatic analysis. The results show that an increase in the section ratio can improve the lateral bearing capacity and energy dissipation capacity of the pier. When the section ratio is increased to 4%, the energy dissipation capacity of a CFST pier is increased by 77.8% and the lateral bearing capacity is increased by 33.9% compared with a section ratio of 2%, but the residual displacement of the pier top also increases. With an increase in the axial compression ratio, the energy dissipation capacity of the pier is significantly improved; when the axial compression ratio is increased to 0.30, the energy dissipation capacity of CFST piers is increased by 27.5% compared with a section ratio of 0.05, the residual displacement of the pier top is reduced, and the self-resetting effect of the pier is improved. A change in the initial prestress has no effect on the energy dissipation capacity of piers. Finally, based on an analysis of mechanical theory, a formula of bending capacity suitable for this type of pier is proposed, and the error is within 10%.

## 1. Introduction

In bridge engineering, the prefabrication and assembly technology of bridge substructures has the characteristics of fast construction and high quality and is also regard as an ecofriendly technology. For urban bridges, the interference with the surrounding environment and traffic during their construction can be reduced. Due to the factory prefabrication, the segmental assembled pier can effectively control the construction quality and quickly install on-site to improve the construction efficiency [1,2]. According to the difference in seismic performance, precast assembled piers can be simply divided into “equivalent cast-in-place” and “non equivalent cast-in-place” [3]. The “non equivalent cast-in-place” precast pier is mainly connected by prestressing tendons. In earthquake events, the precast assembled pier can swing and rotate at the joint, and the provision of restoring force can restore the pier to its original position, effectively reducing the residual displacement of the pier, which ensures the whole structure has a good self-resetting ability [4]. Therefore, a “non equivalent cast-in-place” pier is also called a self-resetting pier and has become a feasible scheme for rapid and green construction that avoids the unacceptable residual deformation of a traditional reinforced concrete pier after an earthquake [5].

Dry joint is one of the common connection methods of precast assembled piers. The longitudinal reinforcement of the pier is discontinuous at the joint, and the concrete sections are connected by post-tensioned prestressing tendons, which have the advantages of convenient construction and easy rapid repair after an earthquake. Due to the poor integrity of the joint, its seismic performance is weaker than that of a cast-in-place pier. Therefore, scholars from various countries have carried out a series of tests and theoretical research. The results show that a precast and assembled pier with dry joints based on the connection of unbonded prestressing tendons has a good self-resetting ability. However, the energy dissipation capacity is poor [6]. The postearthquake damage is mainly the local damage of concrete at the joint of pier bottom. A concrete-filled steel tube (CFST) structure can delay the development of microcracks in core concrete, which avoids local damage to concrete caused by stress concentration during joint opening and closing. Hewes et al. [7] investigated wrapping the pier body with steel pipes in the plastic hinge area at the bottom of the pier to fully utilize the advantages of concrete-filled steel tubes and prevent the damage of the pier in the plastic hinge area. However, it was found that this led to the plastic hinge moving up to the upper segment joint, resulting in serious damage at the segment joint. Based on this, Chou et al. [8] developed a full segmental assembled concrete-filled steel tubular pier in order to reduce the spalling and crushing of the concrete above the bottom segment. However, the pier rotates around the upper and lower joints of the bottom segment under a cyclic load. Gurrini et al. [9] developed a post-tensioned self-resetting segmental assembly of a double-layer segmental assembly of concrete-filled steel tubular piers to reduce the damage of sandwich concrete and reduce the weight of the structure. Hu Liang [10] investigated the seismic behavior of segmental concrete-filled steel tubular piers, the failure mode of segmental concrete-filled steel tubular piers was clarified, and a bi-parametric exponential-type constitutive model of concrete-filled steel tubular piers was proposed. This study provides a reference for the seismic analysis and application of piers.

From the viewpoint of the fracture characterization of heterogeneous materials such as concrete materials, it is widely known that, in the technical literature, several theoretical and numerical methods, based on discrete and smeared damage approaches, have been proposed to predict the damage phenomena with sufficient accuracy, together with the evaluation of crack patterns and crack toughening behavior. ZHAO [11] evaluated the Winfrith model, CDP model, K&C model, and CSC model. The research results showed that the K&C model tends to overestimate the degradation of concrete compressive strength and underestimate the degradation of tensile strength, and no stiffness reduction in either direction was observed in the simulation. Therefore, the K&C concrete model may not be suitable for estimating the cyclic performance of concrete. The Winfrith concrete model cannot reasonably estimate the performance of concrete under repeated loads. The CDP model is in good agreement with the experimental results, and the prediction effect is the best among the four concrete models. Therefore, the CDP model can be used to evaluate the cyclic load of concrete.

For segmental concrete-filled steel tubular piers, the lack of energy consumption capacity is also a serious problem. In recent years, there have been several attempts to study the improvement in the energy dissipation capacity of segmental assembled piers. Jia Junfeng et al. [12] designed experimental models of segmental assembled CFST piers based on post-tensioned prestressing tendon connections and bolt connections. The former has good horizontal bearing capacity and self-resetting capacity, but the pier will have a double plastic hinge effect and a low energy dissipation capacity. The latter, connecting a steel pipe, can improve the energy dissipation capacity and shear resistance of the pier. When the horizontal loading displacement is large, the extrusion between the bolt and the connecting steel pipe will lead to the local yield of the bolt hole, and the residual displacement of the pier top is large. ElGawad [13] designed the pseudostatic test of a double-column self-resetting precast pier with an angled steel damper. The research revealed that the energy dissipation capacity of a prefabricated pier is increased by 75% compared with that of a prefabricated pier without an angled steel damper. The residual displacement is about 10% before the offset rate is 4%. The energy dissipation capacity of a pier is reduced due to the fracture of the angled steel damper, but the concrete pier body is basically undamaged. According to Ichikawa et al. [14], UHPC is used as a precast formwork in the plastic hinge area, in which the reserved hole in the precast formwork passes through the energy dissipation reinforcement, and the other parts are formed by cast-in-place concrete to form the pier as a whole. Finally, the two-way pseudostatic test is used to verify that the UHPC can significantly reduce the damage of the plastic hinge area, and the new precast pier has good seismic performance. Mohebbi et al. [15] investigated a prestressing tendon based on FRP to improve the durability requirements of “non equivalent cast-in-place” precast piers. Tazarv et al. [16] replaced the reinforcement with SMA rods in the plastic hinge area for the bellows grouting connection of the “equivalent cast-in-place” precast pier. Ordinary reinforcement is still used outside the plastic hinge area, and the SMA rods are mechanically connected with ordinary reinforcement. The pseudostatic test research was carried out for this new pier. It was proven that this new pier has a better deformation capacity, basically the same energy dissipation capacity, and the residual deformation is reduced by 79% compared with the cast-in-place pier with the same parameters. Zhang Qiang [17] proposed post-tensioned prestressed segmental precast concrete-filled steel tubular piers and high-strength bolted concrete-filled steel tubular piers, and the force displacement relationship at the top of each precast segment and the moment curvature relationship at the contact surface of each segment were derived. The research was based on ABAQUS numerical analysis and experimental analysis. The result revealed that this pier gives full play to the high performance of concrete-filled steel tubes, improves the energy consumption capacity of segmental assembled piers, avoids the plastic hinge failure of ordinary segmental assembled piers, etc. This technology also improves the production efficiency and can be promoted in high- and medium-seismic-risk areas.

According to the existing research results, the application of concrete-filled steel tubular structures in piers [18] can delay the development of microcracks in the core concrete, avoiding local damage to concrete caused by stress concentration in the process of joint opening and closing. However, the problem of low energy consumption still exists. The bolt hole of the currently used bolt connection is a weak area, which may produce large plastic deformation. SMA shape memory alloy is expensive, and its mechanical properties are easily affected by the environment. Energy-consuming reinforcement is not easy to replace after an earthquake.

**Figure 1:**
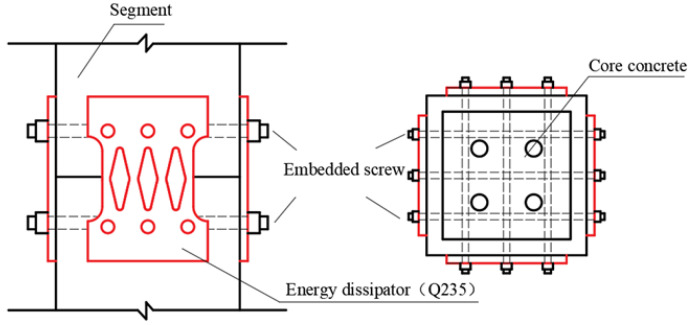
Connection structure of energy dissipation elements.

To solve these problems, this paper proposes a new type of external energy dissipator with a simple structure. This energy dissipator has good shear resistance and energy consumption capacity, is stable and reliable, and is easy to repair and replace after an earthquake. The specific design scheme is shown in Figure 1. CFST pier joints are equipped with energy dissipation devices, which have the following characteristics: During an earthquake, the opening deformation of pier joints can be limited, and plastic deformation can be generated through the device to dissipate energy. The connecting device can provide reliable lateral bearing capacity for the pier and increase the shear stiffness of the pier. Unbonded prestressing tendons provide stable self-resetting ability and reduce the residual displacement of piers. The energy dissipation device gradually narrows the section from the ends to the middle, avoiding the stress concentration at the connection between the device and pier so that the damage is concentrated in the middle part of the energy dissipation device, which is convenient to repair and replace after an earthquake.

## 2. Numerical Simulation and Experimental Verification of Segmental CFST Pier

**Figure 2:**
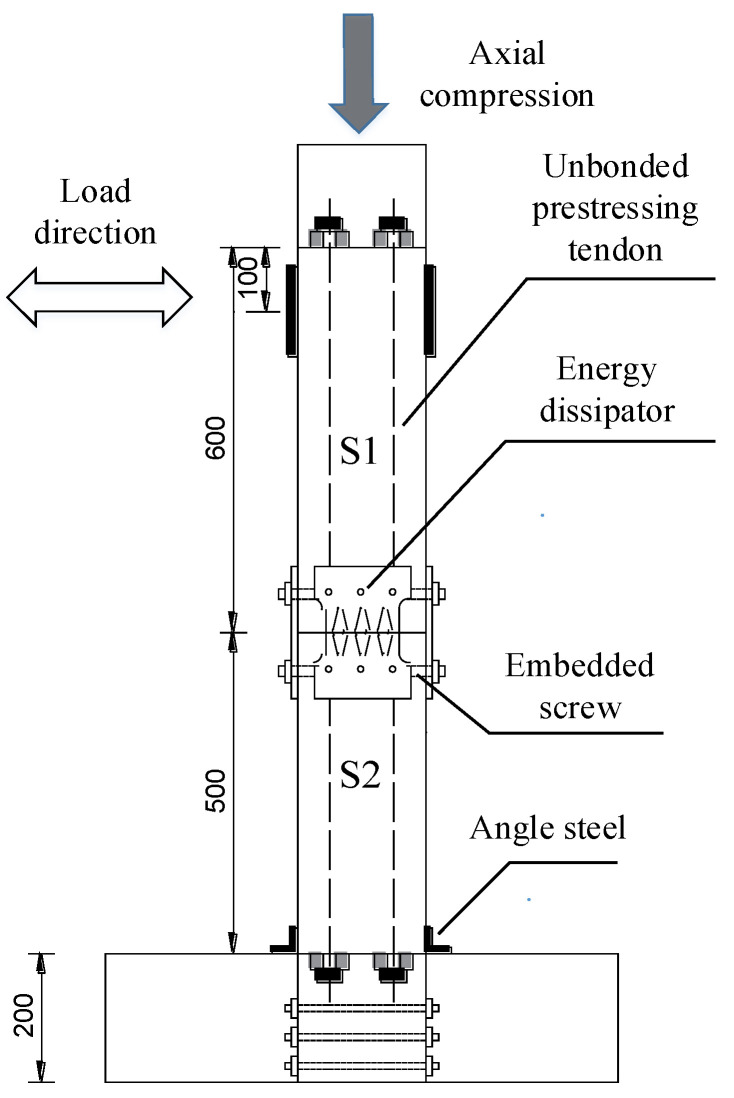
Structure of CFST pier test piece assembled in segments.

In order to verify the feasibility and effectiveness of using finite element analysis, a cyclic loading test of CFST piers with external energy dissipation devices was carried out [19]. The test piece is composed of segmental steel tubes, core concrete, unbonded prestressing tendons, energy dissipation devices, etc. (Figure 1). The pier body of the segmental assembled CFST pier is divided into two segments: S1 and S2. The size of the steel pipe section is l×b×h = 200 mm × 200 mm × 500 mm, with a thickness of 20 mm. Q345 steel is used for steel pipes in sections, and the grade of concrete is C40. Energy dissipation elements are set at the joints of the S1 and S2 segments, which are 60 mm away from the upper and lower ends of the joint. They are connected with S1 and S2 segments through embedded screws, which can not only be used as energy dissipation components but also improve the shear capacity of CFST piers. The energy dissipation element is made of Q235 steel with a thickness of 10 mm, and a diamond hole is opened in the middle, which gradually narrows from both ends to the middle, avoiding the stress concentration damage at the screw hole and ensuring that the weakest position is in the middle of the energy dissipation element. The pier cap and cushion cap pass through 7 *ϕ* 15.2, the unbonded prestressed steel strand is connected, and the applied prestress is 80 kN. The test piece is shown in Figure 2.

**Figure 3:**
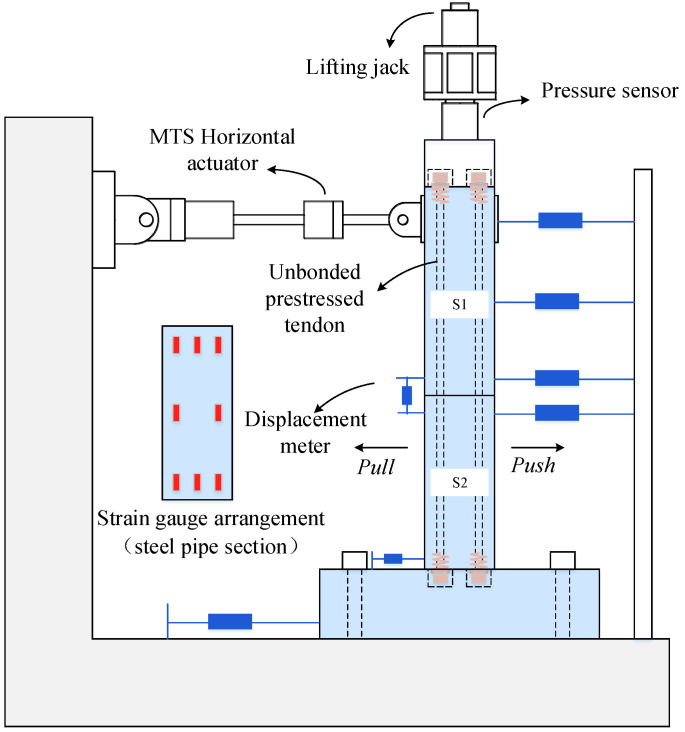
Layout of measuring points.

**Figure 4:**
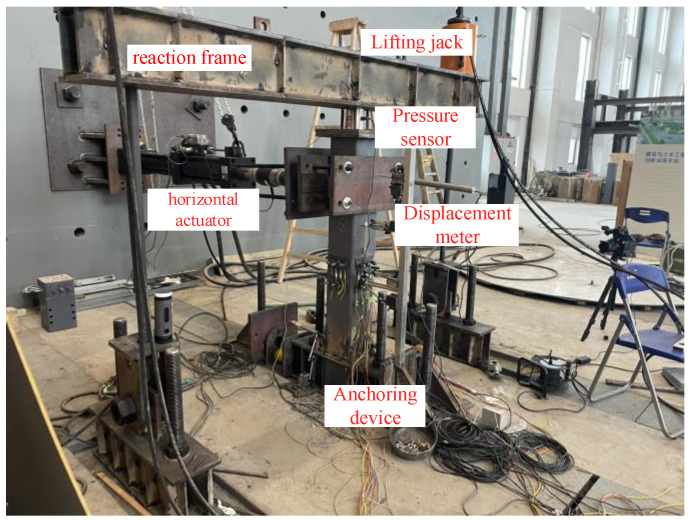
Test loading device.

With an MTS system, this paper carried out the test of the CFST pier, focusing on the nonlinear force displacement relationship, the opening between segments, the residual displacement of segments, and the stiffness degradation under horizontal cyclic loading. The layout of the loading device and the measuring points are shown in Figure 3, and the test loading device is shown in Figure 4.

### 2.1. Material Constitutive

Basic models are established in ABAQUS (6.14, DASSAULT SIMULIA, France). The steel is simulated via various elastoplastic models [20]. The ideal elastoplastic model is for the stress–strain relationship of the energy dissipation elements, steel pipes, and prestressing tendons, which can be expressed as follows:

(1) $$\sigma =\left\{\begin{array}{l} E_{s}\varepsilon ,\varepsilon \leq \varepsilon_{y}\\ f_{y}\;,\varepsilon >\varepsilon_{y} \end{array}\right\}$$

where:

$E_{s}$—Elastic modulus of steel;

$f_{y,\;\;}\;\varepsilon_{y}$—Yield strength and corresponding yield strain of steel;

σ,  ε—Steel stress and corresponding strain.

The yield strength and elastic modulus of steel were measured [21], and the Poisson ratio is 0.3.

The test pier was a concrete-filled steel tubular structure. For the mechanical properties of the test materials to have a great impact on the seismic performance of the pier, compressive and tensile tests were carried out on the concrete. The compressive strength was obtained with an axial loading test according to the GB 50010-2010 [22] standard for test methods of the physical and mechanical properties of concrete. Each group included three concrete test blocks (150 mm cubes), and the results were averaged. The elastic modulus of C40 concrete is 3.18 × 10^4^ N/mm^2^. At the same time, we used three groups of concrete test blocks (150 mm cubes) to carry out the split test. We added a strip between the upper and lower bearing surfaces of the test piece and the press plate so that the test piece could form a corresponding strip load up and down, causing the split failure of the test piece along the cube center or cylinder diameter section. The axial tensile strength of the concrete could be obtained by converting the force value during the split, as shown in Table 1.

The large general finite element program ABAQUS is widely used in the field of seismic analysis of high-rise, long-span building structures and large bridge structures due to its good postprocessing program and powerful nonlinear solver. ABAQUS provides three concrete constitutive models: (1) a brittle cracking model, (2) a dispersion cracking model, and (3) a plastic damage model. The brittle cracking model only considers the nonlinear behavior of concrete in tension, which is suitable for the simulation of the constitutive relationship of concrete materials in plain concrete or less reinforced concrete structural members and is mainly used for the simulation of hydraulic dams and other structures. It is not applicable for the simulation of concrete materials in normal reinforced concrete structures and composite structures. The dispersion cracking model homogenizes the discrete concrete cracks in the actual structural members and simulates the behavior of concrete after cracking by modifying the softening section of the tensile stress–strain relationship curve of concrete. The plastic damage model is suitable for simulating the constitutive relationships of concrete materials under reciprocating loads of structural members and can consider the damage, crack development, crack closure, and stiffness recovery of materials under reciprocating loads.

The dispersion cracking model is more accurate in simulating the behavior of a concrete surface crack and is suitable for analyzing the stress of the constantly changing stress–strain space after concrete cracking. The hysteretic rule of the dispersion cracking model is quite different from the actual behavior of concrete materials and cannot be adjusted, while the hysteretic rule of the plastic damage model is more consistent with reality. Therefore, on the premise that the relevant parameters are set reasonably, the plastic damage model can be used to simulate the mechanical behavior of concrete structures or composite structures under reciprocating loads, while the diffuse crack model is only applicable to the mechanical behavior analysis of structures under monotonic loads. Therefore, we chose the concrete damage plasticity (CDP) model [23].

The concrete is simulated by the concrete damage plasticity (CDP) model, and the plastic parameters of concrete are shown in Table 2, where ψ is the expansion angle, ϵ is the flow potential offset value, $f_{b0}/f_{c0}\;$ is the ratio of biaxial ultimate compressive strength to uniaxial ultimate compressive strength, $K_{c}$ is the invariant stress ratio, and  μ is the viscosity coefficient.

The research shows that when the concrete uses the plastic damage model of concrete provided by finite element ABAQUS, the improvement in its restrained strength can be achieved by determining the yield surface function, but the improvement in the plastic property of concrete cannot be accurately simulated directly by finite element ABAQUS, and the constitutive input of confined concrete into ABAQUS software can make up for this shortage, The confined concrete constitutive model includes the increase in peak strain and the improvement in ductility of the descending section of concrete due to the confinement of steel pipe in the concrete.

**Figure 5:**
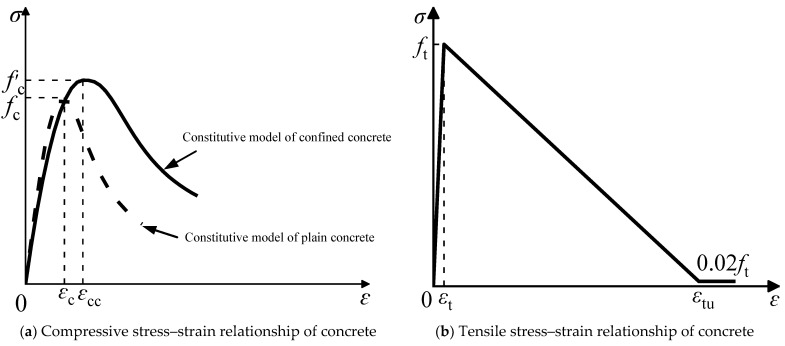
Stress–strain curves of concrete.

Based on this, the confined concrete compression model proposed by Han Linhai [24] was selected as the concrete compression constitutive relationship in the CFST pier model, as shown in Figure 5a, and its expression is as follows:

(2) $$y=\left\{\begin{array}{l} 2x-x^{2},x\leq 1\\ \frac{x}{\beta_{0}{\left(x-1\right)}^{\eta }+x}\;,x>1 \end{array}\right\}$$

(3) $$x=\frac{\varepsilon }{\varepsilon_{0}}$$

(4) $$y=\frac{\sigma }{\sigma_{0}}$$

(5) $$\sigma_{0}=f_{c}{}^{’}$$

(6) $$\varepsilon_{0}=\varepsilon_{c}+800\xi^{0.2}\times 10^{-6}$$

(7) $$\varepsilon_{c}=(1300+12.5f_{c}{}^{’})\times 10^{-6}$$

(8) η=1.6+1.5/x

(9) $$\beta_{0}=\frac{{\left(f_{c}{}^{’}\right)}^{0.1}}{1.2\sqrt{1+\xi }}$$

(10) $$f_{c}{}^{’}=\left[0.76+0.2\log_{10}(\frac{f_{cu}}{19.6})\right]f_{cu}$$

(11) $$E_{c}=4730\sqrt{f_{c}{}^{’}}$$

where $\beta_{0}$ is the adjustment parameters of the descending section of the stress–strain curve of the concrete under compression; η is curve shape coefficient; $\varepsilon_{0},\varepsilon$ are the peak strain of the confined concrete and the concrete strain; $\sigma_{0},\sigma$ are the constraint concrete stress and the concrete stress; $\varepsilon_{c}$ is the peak strain of plain concrete; ξ is the hoop coefficient; $f_{c}^{’}$ is compressive strength of a concrete cylinder; and $E_{c}$ is the elastic modulus of concrete.

The tensile constitutive relationship of concrete is shown in Figure 5b, and its expression is as follows:

(12) $$f_{t}=0.26f_{cu}^{2/3}$$

(13) $$\varepsilon_{t}=65\times 10^{-6}f_{t}^{0.54}$$

(14) $$\varepsilon_{tu}=25\varepsilon_{t}$$

where $f_{t}$ is the tensile strength of concrete; $f_{cu}$ is the concrete cube compressive strength; $\varepsilon_{t}$ is the strain corresponding to the tensile strength of concrete; and $\varepsilon_{tu}$ is the ultimate tensile strain of concrete.

### 2.2. Element Type

Because the continuous element in ABAQUS is not limited by the structure and working conditions, it is mostly used for the three-dimensional simulation of the solid components of the system. In the simulation of segmental assembled CFST piers, the reduced integral element (C3D8R) is used in the solid parts of the pier body (concrete segment, steel pipe, and energy dissipator). In the calculation process, the influence of mesh distortion on displacement accuracy and the influence of shear self-locking on structural stiffness can be avoided [25]. For the model of unbonded prestressing tendons, the truss element (T3D2) was used, which is suitable for simulating slender tension and compression members that only transmit axial force.

### 2.3. Define Contact

In this paper, dry joints are used between pier segments, which open and close repeatedly with cyclic loads in the process of stress, and the relative slip between joints also needs to be considered. Therefore, based on the surface contact algorithm in ABAQUS, surface to surface contact is used for the contact at the joint between segments. The normal behavior adopts the definition of “hard contact” that transfers pressure only when the gap between the contact surfaces is 0, that is, the normal pressure is transferred when the surfaces contact, and the node constraint fails when they are separated. Normal behavior is used to simulate the opening and closing of joints. “Penalty friction” is for the tangential behavior, which defines the interface friction characteristics with the friction coefficient. The friction coefficient is 0.4 [26]. Tangential behavior is used to simulate the relative slip between segments. Tie contact binding is for the contact between steel pipe and concrete, between the energy dissipator and steel pipe, and between the ground beam and the steel pipe section [27].

### 2.4. Boundary Condition

The segmental assembled CFST pier was designed as a cantilever with a consolidated pier bottom and a free pier top. For the convenience of analysis, reference points, RP2 and RP1, were set at the center of the pier bottom and the center of the pier top, respectively, which are coupled with the base and the pier top through coupling constraints. At the same time, the pier bottom consolidation was completed by fully constraining the six degrees of freedom of the reference point RP2, and the reference point RP1 can complete the application of external load and the output of the reaction force and the displacement [28].

### 2.5. Unbonded Prestressed Reinforcement

The joints at both ends of the unbonded prestressed reinforcement are anchored together with the pier top center and the foundation center through an MPC beam restraint. The ideal elastic–plastic model is for the constitutive model of the prestressed reinforcement, and the post-tensioned prestress is applied by the cooling method. The calculation formula is as follows:

(15) $$P=\alpha_{l}\Delta_{t}E_{s}A_{s}$$

where P is the preload applied for post-tensioning; $\alpha_{l}$ is the linear expansion coefficient of the prestressed steel strand; $\Delta_{t}$ is the temperature variable of the temperature field; and $E_{s}\;\mathrm{and}\;A_{s}$ are the elastic modulus and cross-sectional area of the prestressed steel strand, respectively.

### 2.6. Meshing

**Figure 6:**
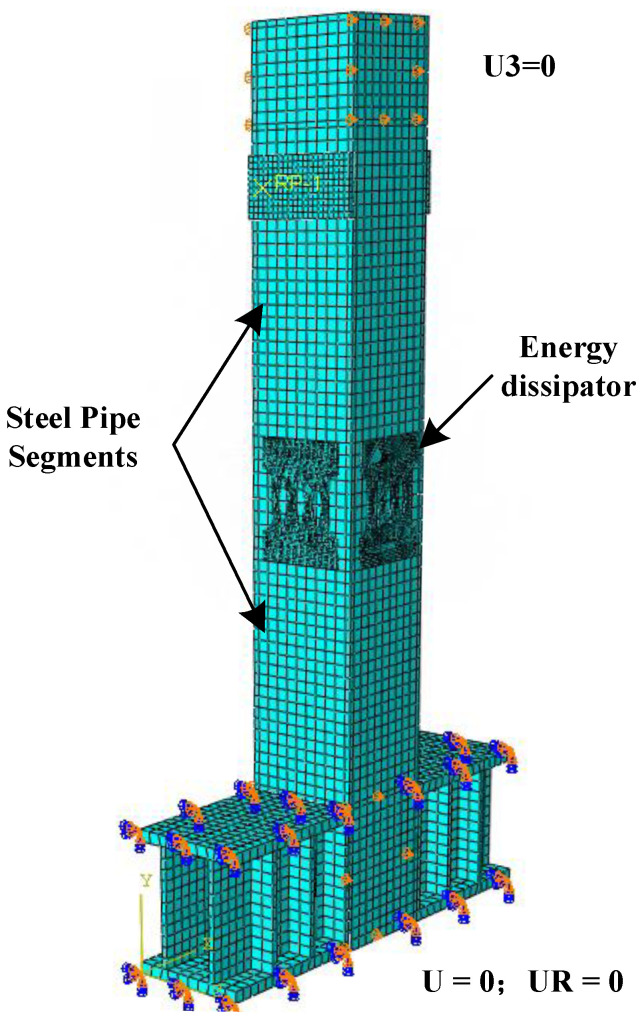
Finite element model of CFST pier.

Finite element meshing is an important part of the model because the number of meshes not only directly affects the accuracy of the calculation results but also affects the calculation efficiency. In order to ensure the convergence and accuracy, the hexahedral mesh and structured mesh technology were divided to obtain better calculation accuracy and reduce the calculation workload. A sensitivity analysis showed that, considering the calculation efficiency and accuracy, the overall mesh density of steel pipe, concrete, and ground beam is 20 mm, the key parts (the connection between the energy dissipator and the steel pipe) are 10 mm, and the mesh density of energy the dissipating elements is 1 mm [29]. The finite model is shown in Figure 6.

### 2.7. Analysis Step and Loading System

In order to make the calculation result closer to the actual force, three analysis steps were set up. In the first analysis step, an axial force of 20 kN was applied on the top of the pier for preloading. The initial increment step was 0.001, the maximum increment step was 0.01, and the maximum increment step was 1000. In the second analysis step, the initial increment step was 0.1, the maximum increment step was 1, and the maximum increment step was 1000. An axial force of 180 kN was applied to the pier top. In the third analysis step, the horizontal load was applied to the top of the pier. The horizontal load is controlled by the displacement and usually has good convergence [30].

**Figure 7:**
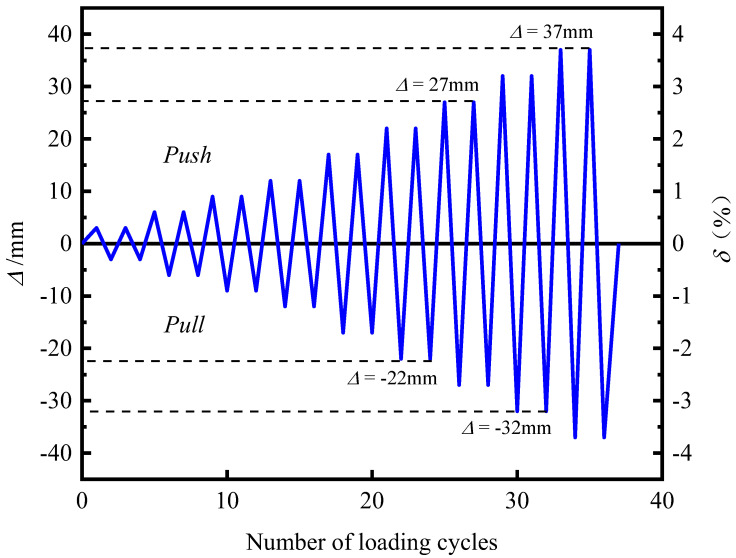
Loading system.

The specimen was loaded with low-cycle cyclic loading. The loading position was 100 mm away from the top of the S1 segment, and the bottom of the pier column was fully restrained to form a cantilever structure. The loading mode was the displacement controlling method. The displacement amplitude of each stage was cycled forward and backward twice, and the displacement offset rates of the loading point were 0.3%, 0.6%, 0.9%, 1.2%, 1.7%, 2.2%, 2.7%, 3.2%, and 3.7%, and the loading displacement increased from 3 mm to 37 mm. The loading scheme is shown in Figure 7.

### 2.8. Solving Method of Nonlinear Equations

ABAQUS finite element analysis involves material nonlinearity and geometric nonlinearity, and nonlinear equations need to be solved in the calculation process. The iterative method, the incremental method, and the incremental iterative method are the main methods to solve nonlinear equations. The incremental iteration method has the advantages of the former methods, so the incremental iteration method is used in this paper. For the incremental method, the automatic incremental method is used. There are three available iterative methods in ABAQUS: The Newton method, the modified Newton method, and the quasi-Newton method. The Newton method has the largest amount of iterative calculation, but it has good convergence and a fast calculation speed. Therefore, the analysis is based on the Newton method in this paper.

### 2.9. Result Analysis

**Figure 8:**
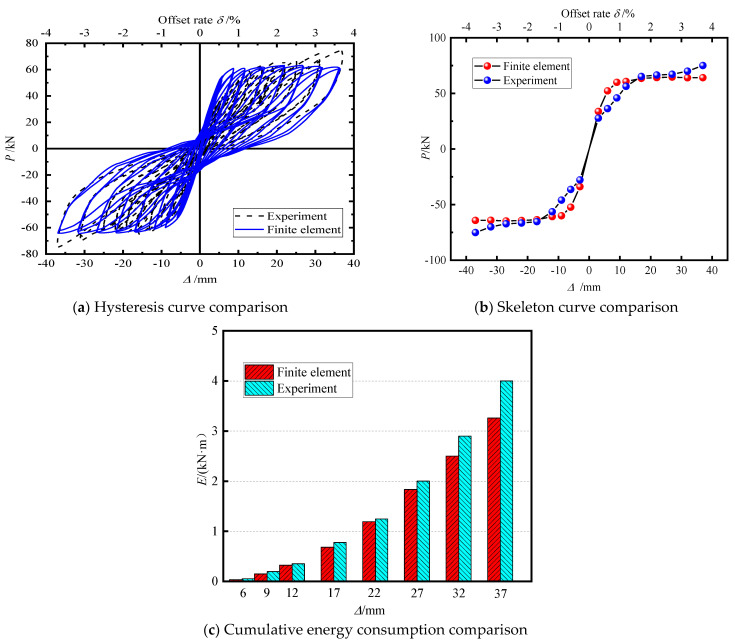
Comparison between experiment and finite element.

Figure 8a shows the hysteretic curve obtained from the test and numerical simulation of segmental assembled CFST piers. When loading, the test was in good agreement with the experimental results. However, with an increase in the loading amplitude, the difference gradually became significant because there was a certain difference between the ground parameters obtained from the simple steel and concrete material test and the real concrete-filled steel tube, which is consistent with the simulation in OPENSEES [31].

Figure 8b shows the skeleton curve comparison between the test and the finite element. It can be seen from the figure that the peak bearing capacity obtained from the initial loading test was slightly lower than that obtained from the finite element, but when the loading displacement reached 17 mm, the curves of the two were basically consistent.

Figure 8c shows the cumulative energy consumption comparison between the test and the finite element. It can be seen from the figure that the results obtained from the initial loading test and the finite element tended to be consistent. When the loading displacement reached 37 mm, there was a slight gap between the two curves, but the error was guaranteed to be about 20%, which is related to the difficulty in controlling the hysteresis curve in the finite element simulation.

Table 3 shows the comparison between the simulation results and the experimental results. Comparing the horizontal bearing capacity, residual displacement, equivalent stiffness, and energy consumption, it can be seen that the numerical simulation was in good agreement with the experimental results. The errors were within a reasonable range, and it had good reliability. The finite element model is suitable for the analysis of the seismic performance of segmental assembled CFST piers with external energy dissipation devices.

## 3. Factors Affecting the Seismic Performance of Segmental CFST Piers

Based on the proposed segmental assembled CFST pier with external energy dissipation devices, the refined model was established by using the numerical simulation method in Section 1. The effects of the axial compression ratio, *a*, initial prestress, *p,* and section ratio, *λ* (the ratio of the cross-sectional area of the energy dissipation elements to the cross-sectional area of the steel pipe sections), on the seismic performance of CFST piers are discussed in the following section. The displacement loading method is shown in Figure 7, with displacement increases from 3 mm to 37 mm. The working condition settings are shown in Table 4.

Ideally, the bigger the section ratio is, the thicker the energy dissipating element is and the better the stiffness and seismic performance of a CFST pier will be. However, when the section ratio is increased to a certain extent, the self-resetting effect of a CFST pier becomes worse. What we are looking for is the best limit that can guarantee the stiffness and seismic performance of a CFST pier as well as have a better self-resetting effect. The axial compression ratio, *a,* refers to the ratio of the design value of axial pressure of a pier column to the product of the design value of the full section area of a pier column and the design value of the axial compressive strength of concrete. It reflects the compression of the pier column; the prestress values of 40 kN, 80 kN, and 120 kN represent 10%, 20%, and 30% of the ultimate strength of the prestressed reinforcement.

### 3.1. Influence Analysis of Section Ratio

In order to study the influence of the section ratio on the seismic performance of segmental CFST piers, the hysteretic curve, cumulative energy consumption, residual displacement of the pier top, and stiffness degradation under a cyclic load were compared (λ = 2%, 3%, and 4%).

**Figure 9:**
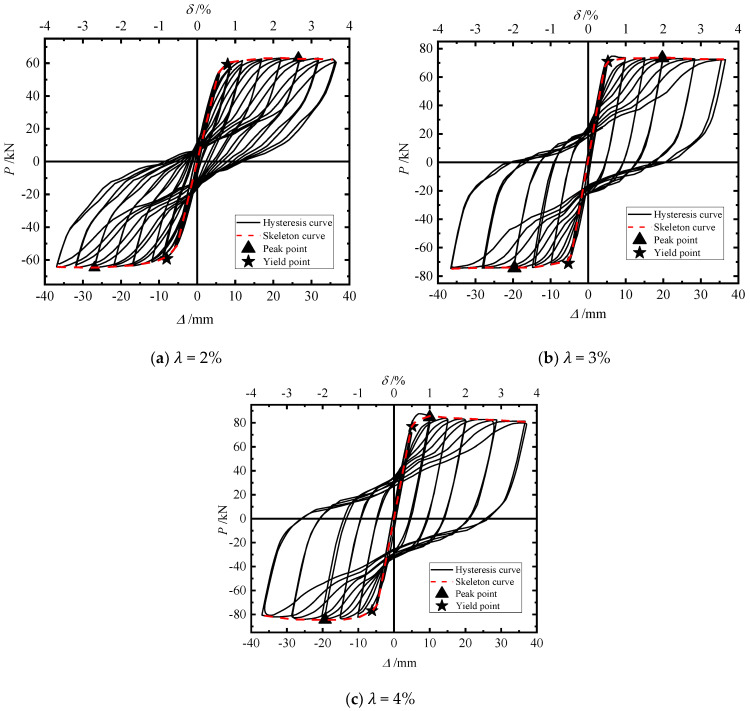
Hysteresis skeleton curve.

Figure 9 shows the hysteretic skeleton curve, and Figure 8a–c correspond to λ = 2%, 3%, and 4%, respectively. The characteristic points of the skeleton curve of the test piece are shown in Table 5, where $P_{y}$: yield load; $\Delta_{y}$: yield displacement; $P_{m}$: peak load; $\Delta_{m}$: peak displacement; $\delta_{u}$: maximum displacement offset rate; and α: displacement ductility coefficient.

It can be seen in Figure 9 that in the initial stage of loading, the slope of the hysteresis curve is large, the residual displacement after unloading is basically zero, and the structure is in an elastic state. With an increase in the loading displacement, the energy dissipation elements yield, the slope of the hysteresis curve decreases, the envelope area of the hysteresis loop increases, and the structure enters the elastic–plastic energy dissipation stage.

Comparing the hysteretic curves of the three models, it can be found that with an increase in the section ratio the shape of the hysteretic curve develops from “flag” to “shuttle”, the hysteretic loop gradually tends to be full, and the energy consumption capacity is further improved. With an increase in the section ratio, the lateral bearing capacity increases significantly because the increase in the section ratio and the increase in the thickness of the energy dissipation element make its own stiffness increase significantly, which can contribute more horizontal force to the structure. Due to the increase in the unrecoverable plastic deformation of the energy dissipation elements, however, the deformation recovery capacity of piers becomes poor, and the residual displacement gradually increases, which makes it more difficult to repair after the earthquake.

It can be seen in Table 5 that, compared with λ = 2%, for λ = 3% and λ = 4% the yield load of the model increased by 20% and 29.78%, respectively, and the peak bearing capacity increased by 16.09% and 33.86%, respectively. It can be seen that the peak bearing capacity and yield load of the model increased with the increase in the section ratio. With the increase in the section ratio, the ductility of the pier first decreased and then increased because the peak displacement and yield displacement of the pier decreased with the increase in the section ratio, but their ranges were not consistent. With the increase in the section ratio, the peak bearing capacity increased, but the peak displacement decreased, which significantly increased the equivalent stiffness. Therefore, increasing the section ratio can enhance the ability of a pier to resist deformation.

**Figure 10:**
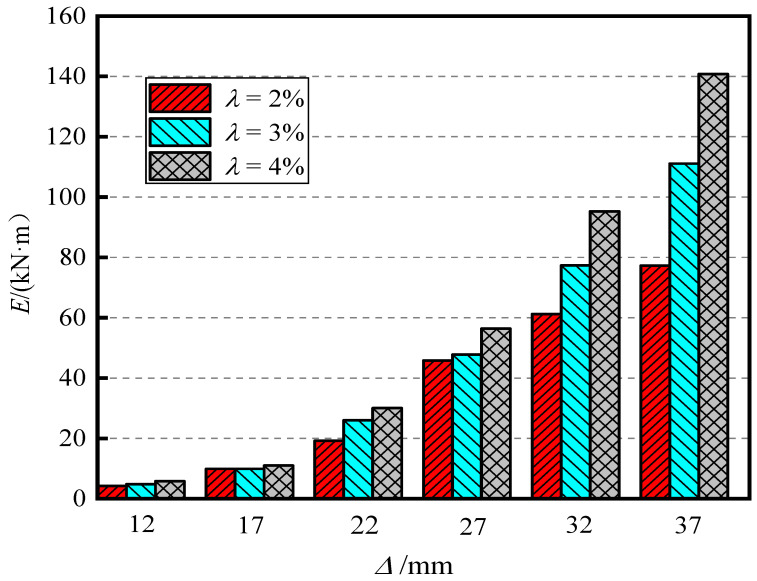
Cumulative energy consumption curve.

Figure 10 shows the influence of the section ratio on the energy dissipation capacity of piers. Through the comparison chart, it can be found that when the displacement loading amplitude is small, the cumulative total energy consumption of the three models is basically the same. When the displacement amplitude exceeds 27 mm (the offset rate is 2.7%), with the increase in the section ratio, the cumulative total energy consumption gradually increases, and the increase also gradually increases. When the displacement amplitude reached 37 mm (3.7% offset), for λ = 2%, 3%, and 4%, the final cumulative energy consumption of the pier was 79.64 kN·m, 112.36 kN·m, and 141.67 kN·m, respectively, which shows that increasing the section ratio helps to improve the energy consumption capacity of the pier.

**Figure 11:**
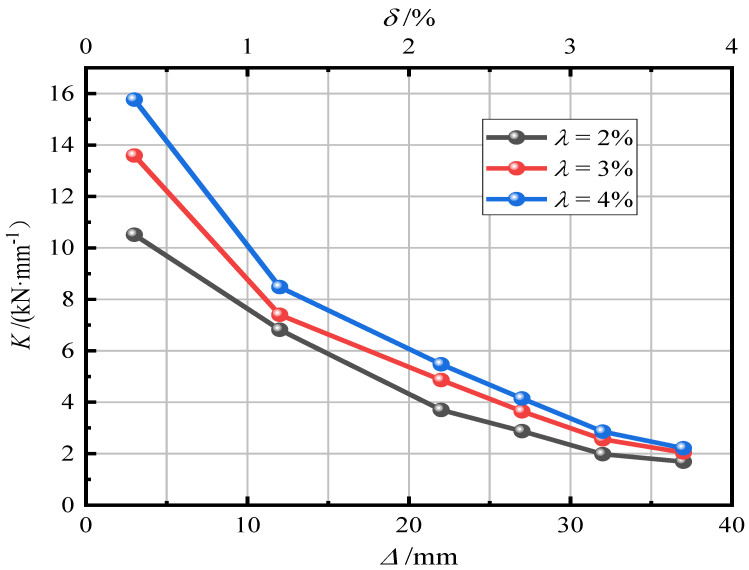
Stiffness degradation law.

Figure 11 shows the effect of the section ratio on pier stiffness degradation. It can be seen that an increase in the section ratio can improve the initial stiffness of the pier, restrain the opening of the joint surface of the segment, effectively reduce the rotation deformation of the rigid body, and make the stress characteristics at the joint surface gradually close to the cast-in-place pier column so as to enhance the ability of the pier to resist deformation. With an increase in the section ratio, the degradation rate of stiffness gradually increases. When the displacement amplitude increases, the gap of the equivalent stiffness curve gradually decreases and finally tends to be consistent.

**Figure 12:**
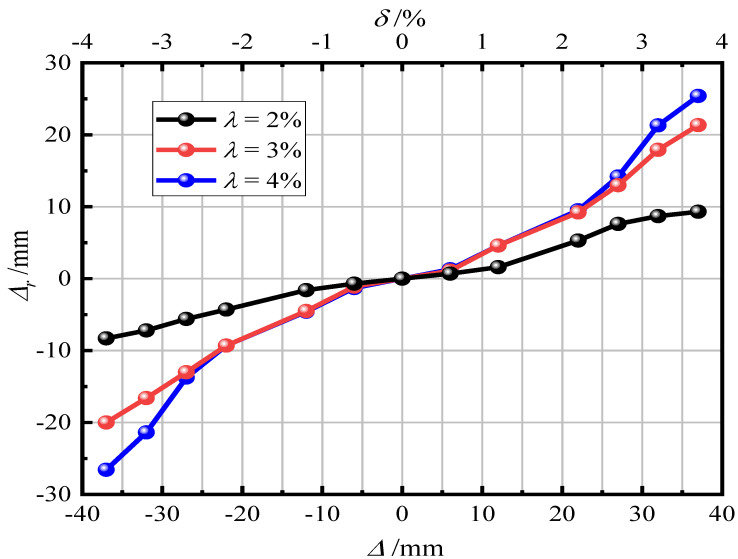
Residual displacement.

Figure 12 shows the influence of the section ratio on the residual displacement of the pier. It can be seen in Figure 11 that the when the section ratio was relatively small, the residual displacement was relatively small and the self-resetting ability was stronger. With an increase in the loss section ratio, the residual displacement increased continuously. After loading, for λ = 2%, 3%, and 4%, the residual displacement was 8.93 mm (0.89%), 22.52 mm (2.25%), and 27.35 mm (2.74%) respectively. This shows that an increase in the section ratio makes the self-resetting ability of the pier gradually worse. In the existing analysis, it is suggested that the section ratio should not exceed 2%.

### 3.2. Influence Analysis of Axial Compression Ratio

In order to study the influence of the axial compression ratio on the seismic performance of segmental CFST piers, the seismic performance of piers under cyclic loading, such as the hysteretic curve, cumulative energy consumption, residual displacement of the pier top, stiffness degradation, etc., were compared at a = 0.05, 0.15, and 0.30.

**Figure 13:**
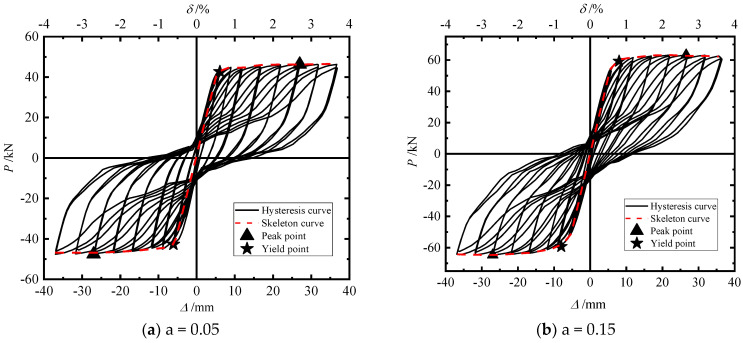

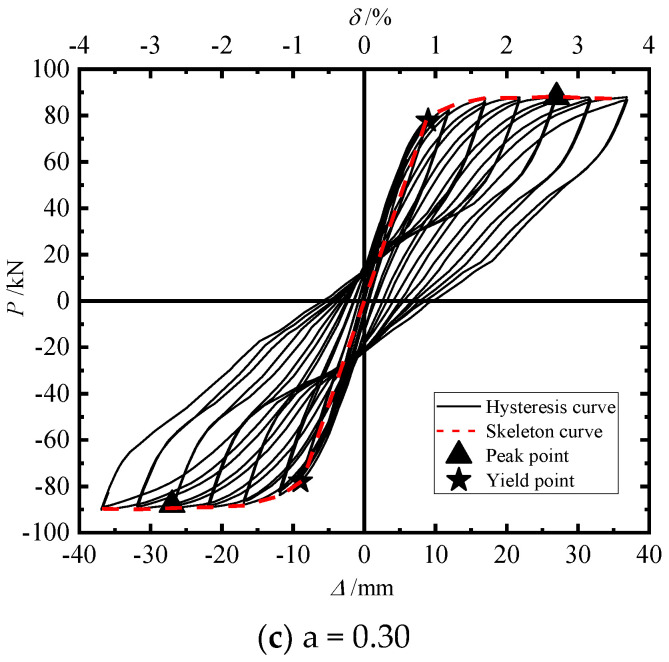
Hysteresis skeleton curve.

Figure 13 shows the hysteretic skeleton curves, and Figure 12a–c correspond to a = 0.05, 0.15, and 0.30, respectively. The characteristic points of the skeleton curve of the test piece are shown in Table 6, where $P_{y}$: yield load; $\Delta_{y}$: yield displacement; $P_{m}$: peak load; $\Delta_{m}$: peak displacement; $\delta_{u}$: maximum displacement offset rate; and α: displacement ductility coefficient.

Comparing the hysteretic curves of the three models, it can be found that with an increase in the axial compression ratio, the lateral bearing capacity of the model gradually increases, which is because the increase in the axial compression ratio makes the connection between segments more compact, so that the pier can provide greater horizontal force. The shapes of the hysteresis curves of the three models are basically the same, showing a “flag” shape, and the pinch effect is obvious. In the early stage of loading, the smaller the axial compression ratio, the fuller the hysteresis loop. After the energy dissipation element yields (the loading displacement reaches 27 mm), the larger the axial compression ratio, the fuller the hysteresis loop. On the whole, an increase in the axial compression ratio has a significant increase when the loading displacement is large.

The characteristic points of the skeleton curve of the test piece are summarized in Table 6. Compared with a = 0.05, when a = 0.15 and a = 0.30 the yield load of the model increased by 38.8% and 82.38%, respectively, and the peak bearing capacity increased by 32.82% and 84.8%, respectively. It can be seen that the peak bearing capacity and yield load of the model increased with an increase in the axial compression ratio. With an increase in the axial compression ratio, the peak bearing capacity increased, but the peak displacement was basically the same, which makes the equivalent stiffness increase significantly. Therefore, increasing the axial compression ratio can enhance the ability of a pier to resist deformation. When the axial compression ratio increased from 0.05 to 0.30, the ductility of the pier decreased from 4.38 to 3.02, a decrease of 31.05%, which indicates that an increase in the axial compression ratio will reduce the ductility of the pier.

**Figure 14:**
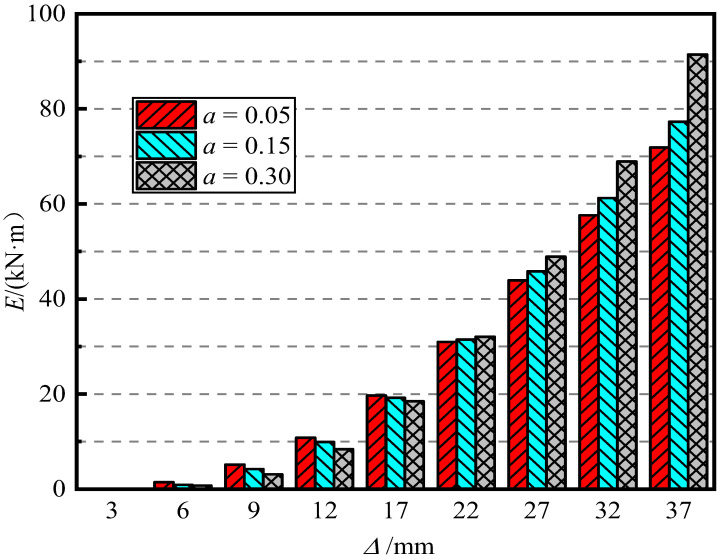
Cumulative energy consumption.

Figure 14 shows the influence of the axial compression ratio on the energy dissipation capacity of piers. It can be seen in Figure 14 that when the loading displacement is small, that is, the loading displacement is less than 22 mm (the offset rate is less than 2.2%), the energy dissipation capacity of the pier decreases with an increase in the axial compression ratio, but it gradually tends to be consistent. When the displacement amplitude reaches 37 mm (offset rate 3.7%), for a = 0.05, 0.15, and 0.30, the final cumulative energy consumption of the pier was 72.53 kN·m, 79.64 kN·m, and 92.47 kN·m, respectively. It can be seen that an increase in the axial compression ratio improves the energy consumption capacity of the pier.

**Figure 15:**
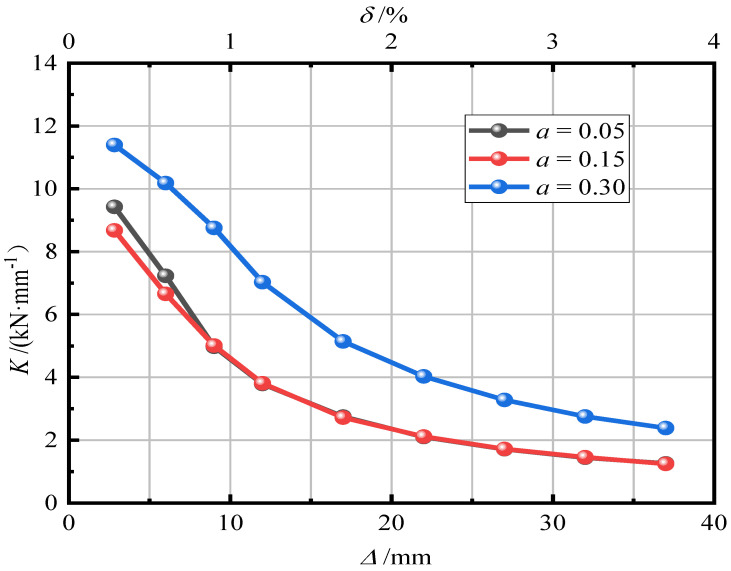
Stiffness degradation law.

Figure 15 shows the effect of the axial compression ratio on pier stiffness degradation. It can be seen from Figure 14 that when the axial compression ratio was 0.05 and 0.15, the two curves were similar. When the axial compression ratio was 0.30, the initial stiffness of the pier significantly improved, indicating that when the axial compression ratio increases to a certain value, the ability of the pier to resist deformation can be improved. The rate of stiffness degradation was basically the same. An increase in the axial compression ratio will accelerate the rate of stiffness degradation, and the rate of stiffness degradation will gradually flatten after the specimen reaches yield.

**Figure 16:**
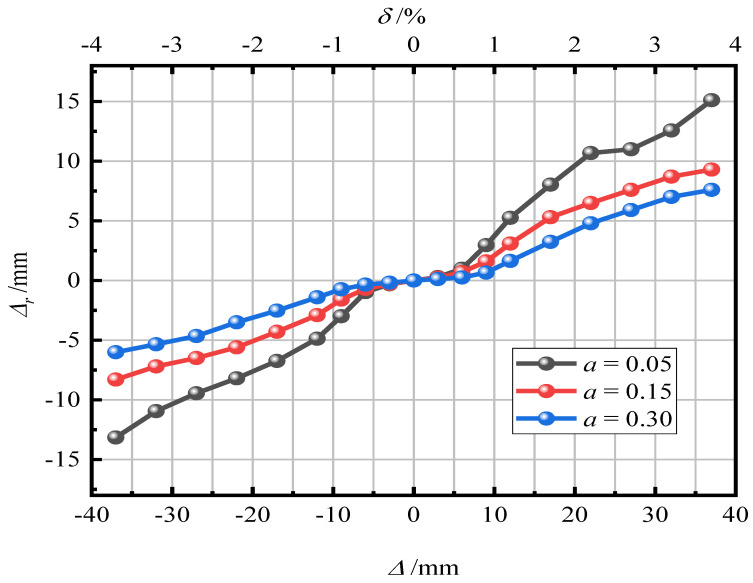
Residual displacement.

Figure 16 shows the influence of the axial compression ratio on the residual displacement of the pier. It can be seen in Figure 15 that with an increase in the axial compression ratio, the residual displacement of piers gradually decreases. After loading, the residual displacement of piers with a = 0.05, 0.15, and 0.30 was 15.12 mm (1.51%), 8.93 mm (0.89%), and 6.53 mm (0.65%), respectively. It reveals that an increase in the axial compression ratio can improve the self-resetting ability of piers. The larger the axial compression ratio, the smaller the residual displacement of piers and the stronger the self-resetting ability of piers.

### 3.3. Influence Analysis of Initial Prestress

In order to investigate the influence of initial prestress on the seismic performance of segmental assembled CFST piers, the seismic performance of piers under cyclic loads (*p* = 40 kN, 80 kN, and 120 kN) was compared, including the hysteretic curve, cumulative energy consumption, residual displacement of the pier top, and stiffness degradation.

**Figure 17:**
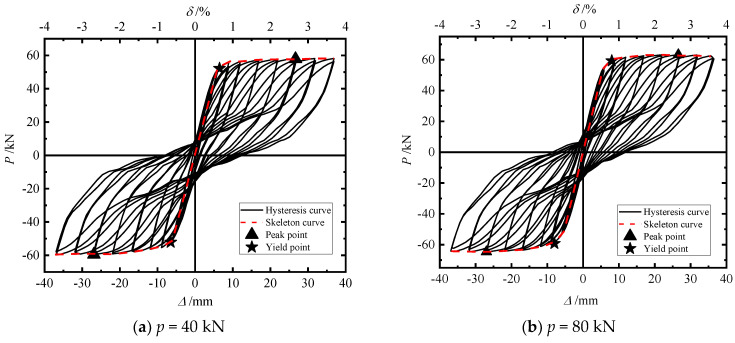

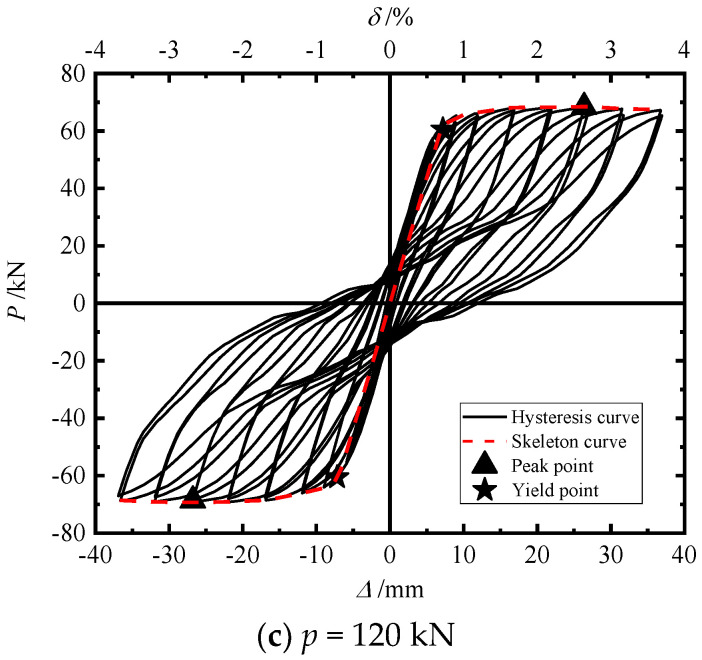
Hysteresis skeleton curve.

Figure 17a–c correspond to p = 40 kN, 80 kN, and 120 kN, respectively. The characteristic points of the skeleton curve of the test piece are summarized in Table 7, where $P_{y}$: yield load; $\Delta_{y}$: yield load; $P_{m}$: peak load; $\Delta_{m}$: peak displacement; $\delta_{u}$: maximum displacement offset rate; and α: displacement ductility coefficient.

Comparing the three hysteresis curves, it can be found that the hysteresis curves are “flag” shaped, the hysteresis loops are relatively full, and the pinch effect is obvious, indicating that they all have good energy consumption capacity. With an increase in initial prestress, the lateral bearing capacity of the pier was further improved, the envelope area of the hysteretic curve gradually increased, and the energy dissipation capacity of the pier also improved.

It can be seen in Table 7 that compared with *p* = 40 kN, for *p* = 80 kN and *p* = 120 kN the yield load of the model increased by 13.59% and 16.01% and the peak bearing capacity increased by 9.28% and 18.17%, respectively. The peak bearing capacity and yield load of the model increased with an increase in the initial prestress, but the increase was not obvious. The displacement ductility coefficient of the pier decreases with an increase in the initial prestress. The initial prestress increased by 200%, and the ductility coefficient of the pier decreased by 27.6%. With an increase in initial prestress (200%), the peak bearing capacity increased (18.2%), but the peak displacement decreased slightly (1.1%), which increased the equivalent stiffness of the pier. Therefore, increasing the initial prestress can enhance the ability of a pier to resist deformation. This may be because the increase in prestress makes the connection between segments more closely so as to improve the stiffness of the pier as a whole.

**Figure 18:**
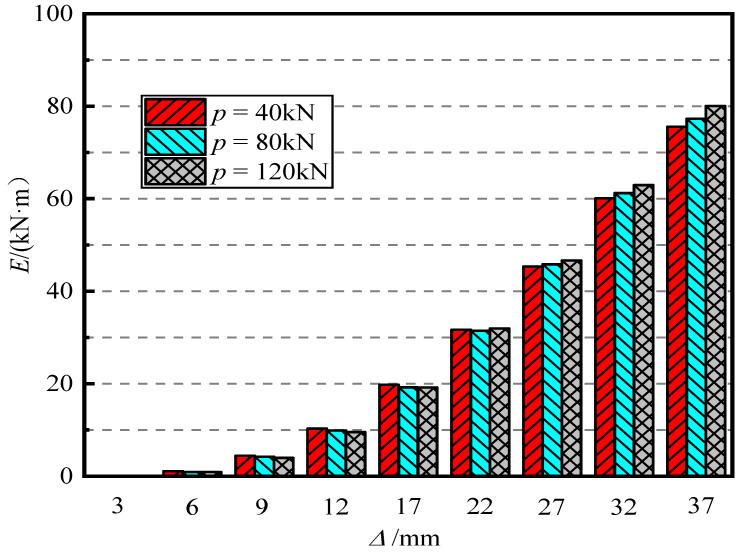
Cumulative energy consumption.

Figure 18 shows the effect of initial prestress on the energy dissipation capacity of piers. It can be seen that the cumulative total energy consumption of piers under different prestressing forces basically tends to be the same. However, when the loading displacement is 37 mm (offset rate 3.7%), the energy consumption capacity increases with an increase in initial prestress. Therefore, changing the initial prestress does not significantly improve the energy dissipation capacity of the pier.

**Figure 19:**
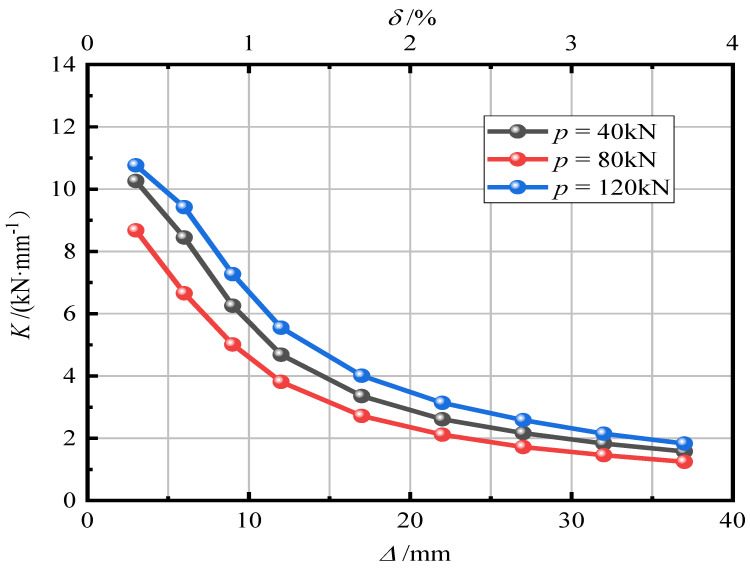
Stiffness degradation law.

Figure 19 shows the effect of initial prestress on pier stiffness degradation. It can be seen in Figure 18 that when the initial prestress is large, the initial stiffness of the segmental assembled CFST pier is high, which indicates that increasing the initial prestress can enhance the lateral bearing capacity and deformation resistance of the pier. The change in initial prestress has little effect on the degradation rate of pier stiffness, but it can be seen in Figure 18 that when *p* = 120 kN the degradation rate of pier stiffness is faster, and the three curves are similar when the loading displacement reaches 37 mm (offset rate 3.7%).

**Figure 20:**
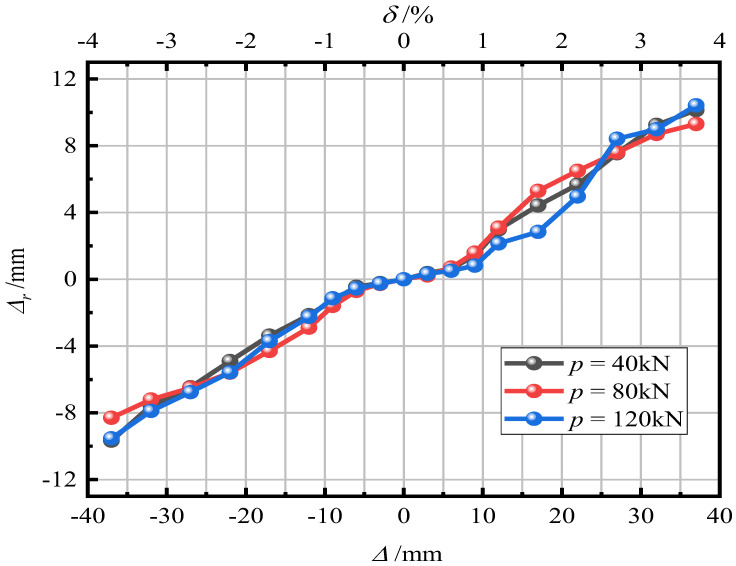
Residual displacement.

Figure 20 shows the effect of initial prestress on the residual displacement of piers. The three curves are similar, which shows that a change in the initial prestress has little impact on the residual displacement of the pier and does not improve the self-resetting ability of the pier.

## 4. Bending Capacity Formula Based on Mechanical Theory Analysis

Due to the existence of joints in precast segmental concrete bridges, the initial fabrication of shear transmission is different from that of conventional bridges. The shear resistance of joints has become an important factor restricting the overall mechanical performance of precast segmental concrete bridges. The last thing we want is shear failure. Only when it is determined that no shear failure has occurred on the piers can it be shown that the main bending deformation of the piers occurs, and the formula of bending bearing capacity of a bridge pier is further deduced.

The shear capacity of precast segmental piers depends on the mutual friction between the concrete at the joints. Buyukozturk, Bakhoum, etc., put forward the calculation formula on the shear performance of precast concrete segmental pier joints [32]. The formula for plane dry joints is as follows:

(16) $$V_{j}=A_{j}\mu \sigma_{n}$$

where:

$V_{j}$—Shear bearing capacity of the joint surface;

$A_{j}$—Contact area of the joint surface;

μ—Friction coefficient;

$\sigma_{n}$—Normal stress value at the joint surface.

The shear capacity of the pier proposed in this paper is not only provided by the mutual friction between the concrete at the joint but is also provided by external energy dissipation devices. Therefore, the above Formula (12) is supplemented as follows:

(17) $$V_{j}=A_{j}\mu \sigma_{n}+nA_{k}f_{v}$$

where:

$A_{k}$—Cross-sectional area of the energy dissipator;

$\;f_{v}$—Shear strength of energy dissipator steel;

n—Number of energy dissipators;

$\;\sigma_{n}$—Provided by axial pressure and prestress;

μ—According to AASHTO American specification [33], μ is 0.6.

**Figure 21:**
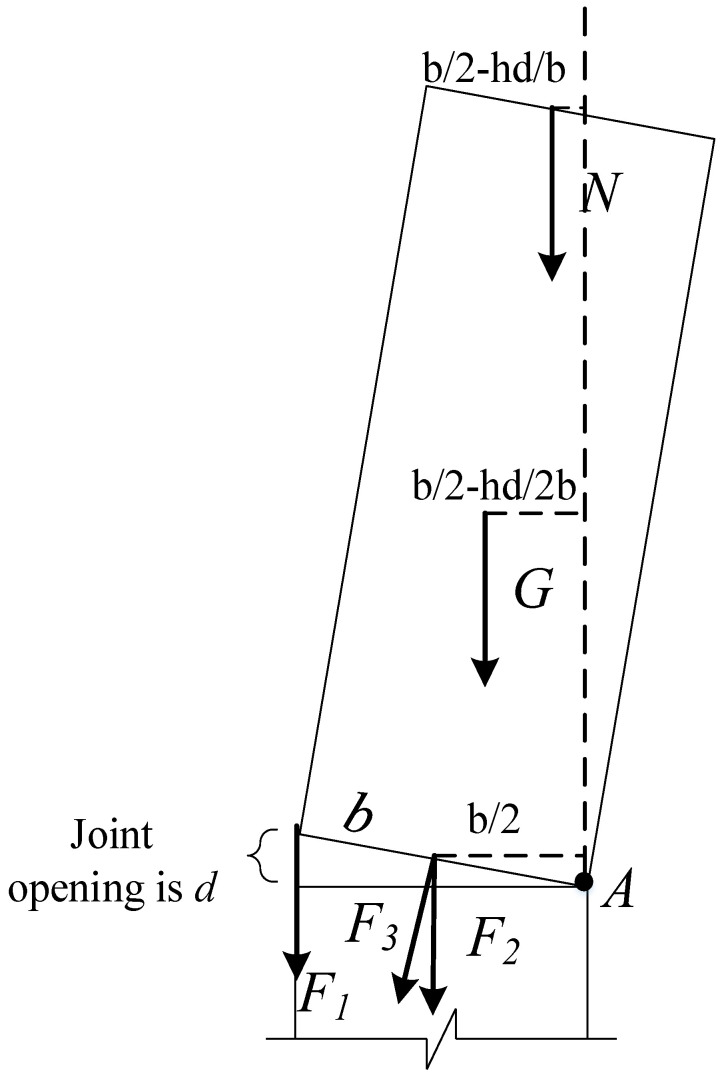
Ultimate stress state of pier bending.

The data of the low-cycle cyclic test of the CFST pier were substituted into Formula (13). $V_{j}$ = 162 kN, which is much larger than the ultimate load of 79 kN in the test. It shows that the pier specimen did not undergo shear failure in the low-cycle cyclic test, so the CFST pier is still in the bending deformation state. By analyzing the stress under a limit state (Figure 21, the moment at point A is calculated), the reference Formula (14) for the bending bearing capacity of a CFST pier suitable for external energy dissipators can be obtained.

(18) $$M_{u}=F_{1}b+\frac{F_{2}+F_{3}}{2}b+G(\frac{b}{2}-\frac{hd}{2b})+N(\frac{b}{2}-\frac{hd}{2b})$$

where:

$F_{1},\;\;F_{2}$—Force of energy dissipators at different positions acting on pier segments;

$F_{3}$—The tensile force produced by the prestressing tendons during the initial prestress and the rotation of the pier segment;

G—The dead weight of the concrete-filled steel tubular section was calculated to be 20 kN;

N—Axial force;

b—Section width of concrete-filled steel tube section;

d—Joint opening (*d* = $\varepsilon_{y}$*l*, where *l* is the length of the energy-consuming element);

h—Height of the steel pipe segment.

By carrying out a one-step derivation of Formula (14), Formula (15) can be obtained:

(19) $$\begin{array}{c} F_{1}=2F_{2}=f_{y}\lambda A\\ F_{3}=p+\frac{1}{2}\varepsilon_{y}E_{s}A_{s}\\ N=aN_{0}\\ M_{u}=f_{y}\lambda Ab+\frac{f_{y}\lambda A/2+p+\varepsilon_{y}E_{s}A_{s}/2}{2}b+G(\frac{b}{2}-\frac{h\varepsilon_{y}l}{2b})+N(\frac{b}{2}-\frac{h\varepsilon_{y}l}{b}) \end{array}$$

where:

$f_{y}$—Yield strength of energy dissipator steel, taken as 235 MPa;

λ—Section ratio;

A—Section area of concrete-filled steel tubular section;

a—Axial compression ratio;

$N_{0}$—Ultimate bearing capacity of the concrete-filled steel tubular pier;

p—Initial prestress;

$\varepsilon_{y}$—Yield strain of energy dissipator steel;

$E_{s}$—Elastic modulus of prestressed reinforcement, taken as 1.95 GPa;

$A_{s}$—Cross-sectional area of prestressed reinforcement, taken as 139 mm^2^.

Substituting the finite element parameters into Formula (15), it can be found that the average value of the ratio between the simulated value and the calculated value of the theoretical formula and the seven finite element models is 0.94. The specific finite element model parameters and the comparison between the simulated and calculated values are summarized in Table 8. It can be seen that the error between the calculated value and the simulated value is basically within 10%, and the accuracy of the formula is high, which can be used as a reference for engineering practice.

## 5. Conclusions

(1) This paper proposes a new type of energy dissipator with a simple structure and a stable energy consumption capacity that is easy to repair and replace after an earthquake. It can also be used as a reference for prefabricated CFST piers.

(2) Based on ABAQUS, the finite element model of a segmental assembled concrete-filled steel tubular pier with replaceable energy dissipators is established. The comparison with the existing experimental results shows that the solid model numerical simulation method designed in this paper can reliably predict the mechanical properties of segmental CFST piers.

(3) An increase in the section ratio can improve the lateral bearing capacity, energy dissipation capacity, and stiffness of the pier, but the residual displacement of the pier top also increases, which makes it difficult to repair after earthquake events. In order to ensure that the segmental CFST pier has good self-resetting and energy dissipation capacity, it is suggested that the section ratio of the energy dissipator should not exceed 2%. With an increase in the axial compression ratio, the energy dissipation capacity of the pier is significantly improved, and the residual displacement of the pier top is reduced. The self-resetting effect of the pier is improved, but the ductility of the pier is reduced. A change in the initial prestress has no effect on the energy dissipation capacity of the pier, and the ductility of the pier deteriorates with an increase in the initial prestress.

(4) Based on the analysis of mechanical theory, the bending bearing capacity formula suitable for this pier was determined, and the error was less than 10%. The formula has high accuracy and can be popularized for similar piers and can be used as a reference for practical projects.

## Figures and Tables

**Table 1:** Axial compressive strength of concrete.

Specimen	1	2	3	Average
Compressive strength (MPa)	42.8	43.1	41.6	42.5
Tensile strength (MPa)	2.35	2.43	2.40	2.39

**Table 2:** Parameters of ABAQUS concrete plastic model.

ψ	ϵ	$f_{b0}/f_{c0}$	$K_{c}$	μ
30	0.1	1.16	0.6667	0.0005

**Table 3:** Comparison between test values and simulation values.

Comparison Item	Horizontal Bearing Capacity/kN Side Shift 6.2%	Residual Displacement/mm Side Shift 6.2%	Equivalent Stiffness/(kN·mm^−1^) Side Shift 6.2%	Energy Consumption/(kN·mm) Side Shift 6.2%
Experimental result	74.1	5.8	1.2	3.3
Finite element results	62.6	8.2	1.0	4.0
Rate	0.8	1.4	0.8	1.2

**Table 4:** Working Condition Settings.

Parameter	Value Range	Invariant Parameter
λ	2%, 3%, 4%	*a* = 0.15, *p* = 80 kN
*a*	0.05, 0.15, 0.30	λ = 2%, *p* = 80 kN
*p*	40 kN, 80 kN, 120 kN	*a*=0.15, λ = 2%

**Table 5:** Characteristic points of skeleton curve of test piece.

λ **/%**	$P_{y}$ **/kN**	$\Delta_{y}$/mm	$P_{m}$/kN	$\Delta_{m}$/mm	$\delta_{u}$/%	α
2	59.24	8.02	63.20	26.58	3.62	3.31
3	71.12	5.78	73.37	19.75	3.66	3.42
4	76.88	5.69	84.60	19.30	3.67	3.39

**Table 6:** Characteristic points of skeleton curve of test piece.

a	$P_{y}$/kN	$\Delta_{y}$/mm	$P_{m}$/kN	$\Delta_{m}$/mm	$\delta_{u}$/%	α
0.05	42.68	6.09	47.58	26.69	3.65	4.38
0.15	59.24	8.02	63.20	26.58	3.62	3.31
0.30	77.84	8.96	87.93	27.03	3.67	3.02

**Table 7:** Characteristic points of skeleton curve of test piece.

*P*/kN	$P_{y}$/kN	$\Delta_{y}$/mm	$P_{m}$/kN	$\Delta_{m}$/mm	$\delta_{u}$/%	α
40	52.15	6.49	57.83	26.67	3.65	4.11
80	59.24	8.02	63.20	26.58	3.62	3.31
120	60.55	8.18	68.34	26.37	3.67	3.22

**Table 8:** Comparison between simulated values and calculated values of flexural bearing capacity.

Model Number	Section Ratio	Axial Compression Ratio	Initial Prestress (kN)	Analog Value $M_{u,e}$ (kN·mm)	Calculated Value $M_{u,c}$ (kN·mm)	$M_{u,e}/M_{u,c}$
1	2%	0.05	80	23.8	27.1	0.878
2	2%	0.15	80	31.6	31.1	1.016
3	2%	0.30	80	38.9	37.1	1.048
4	2%	0.15	40	32.9	35.4	0.929
5	2%	0.15	120	38.2	43.4	0.880
6	3%	0.15	80	36.7	40.1	0.915
7	4%	0.15	80	42.3	45.9	0.921
